# Editorial: Reviews in pharmacology of infectious diseases: 2022

**DOI:** 10.3389/fphar.2023.1236754

**Published:** 2023-07-13

**Authors:** Hong Zhou, Esther Segal, Linda Boniface Oyama

**Affiliations:** ^1^ Key Laboratory of Basic Pharmacology of Ministry of Education and Joint International Research Laboratory of Ethnomedicine of Ministry of Education, Zunyi Medical University, Zunyi, Guizhou, China; ^2^ Department of Microbiology and Clinical Immunology, Sackler Faculty of Medicine, Tel Aviv University, Tel Aviv, Israel; ^3^ Institute of Global Food Security, School of Biological Sciences, Queen’s University Belfast, Belfast, United Kingdom

**Keywords:** infectious diseases, bacterial resistance, antibiotics, antibacterial sensitizers, re-evaluation

Due to the widespread use and abuse of antibiotics, resistance to the effects of bacterial drugs is becoming increasingly serious (Ali et al., 2008; Antimicrobial Resistance Collaborators, 2022). Therefore, rational use of antibiotics and updating research strategies for new drugs are very important.

Antibacterial drugs are important resources to ensure human health. Beta-lactams remain the cornerstone of empirical therapy for various bacterial infections. Haseeb et al. systematically reviewed and analyzed the data describing the dosing regimen of β-lactams. Results showed appropriate antibiotic therapy to be challenging due to pathophysiological changes among different age groups. Optimization of pharmacokinetic/pharmacodynamic (PK/PD) parameters is useful to support alternative dosing regimens, such as an increase in the dosing interval, continuous infusion, and an increased dose of bolus.

The rational use of antibiotics is dependent upon clinicians, but the strong cooperation of clinical pharmacists is also important because it can improve the use of antibacterial drugs and reduce the risk of adverse reactions and bacterial resistance. Du et al. conducted a prospective, historical, controlled study based on pharmacist early active consultation (PEAC) in patients suffering from infection by multidrug-resistant organisms (MDROs). The retrospective control group was comprised of patients hospitalized 18 months before PEAC initiation. The prospective PEAC group was comprised of patients hospitalized 18 months after PEAC initiation. The primary endpoint was 30-day all-cause mortality. Secondary outcomes were MDRO clinical outcome, duration of antibiotic use, duration of hospital stay, antibiotic consumption, and antibiotic costs. Subgroup analysis of secondary outcomes focused on the condition upon hospital admission, MDRO pathogenicity, and MDRO clinical outcome. Results showed that PEAC could reduce 30-day all-cause mortality and improve clinical outcomes in patients infected by MDROs.

Due to the extensive resistance of bacteria to antibiotics, in addition to the rational use of antibiotics, “antibacterial sensitizers” that can be used in combination with antibiotics to reduce or reverse bacterial resistance have become “research hotspots.” Wang et al. postulated that a combination of other compounds with aminoglycoside antibiotics could reduce bacterial resistance effectively and restore the antibacterial activity of aminoglycoside antibiotics.

Against common bacteria and infection induced by *Helicobacter pylori*, which can cause peptic ulcers, gastric ulcers, and gastric cancer, combined use of antibiotics and non-antibiotic drugs (e.g., proton pump inhibitors) can elicit greater efficacy than the use of a single antibiotic alone. To overcome the challenges associated with antibiotic therapy, the biopharmaceutical principles of antibiotics (e.g., PK/PD studies and microbiology) must be considered. Despite challenges, hope exists for improving antibiotic therapy in the near future. Miri et al. provided a survey of curative options based on polypharmacy by looking at concepts, such as PK, PD, and pharmaceutical microbiology, in the battle against infection induced by *H. pylori*. They claimed that there is an urgent need to discover alternative antibiotics for rescuing clinical failures. In one study on a new regimen, Jin et al. (2018) suggested that standard triple therapy containing ilaprazole could be used for the future treatment of *H. pylori* infection.

Natural plants are considered to be a huge “treasure trove” of antibacterial sensitizers, and many antibacterial sensitizers under study are derived from plants. Li et al. postulated that the compounds from plants, such as epigallocatechin gallate, which is the major catechin present in *Camellia sinensis* (L.) Kuntze tea leaves, potentiated the activity of β-lactams against methicillin-resistant *Staphylococcus aureus*. One possible mechanism was the inhibition of β-lactamases in *S. aureus* and interference with cell-wall integrity through direct binding to peptidoglycan.

Bacterial infections are related to the number and virulence of bacteria, but also closely related to the immune status of the human body. Improving the immune system helps to resist the infection caused by common microorganisms, but also improves the resistance to the infection induced by drug-resistant bacteria. Zhu et al. discovered that polysaccharides and flavonoids from *Phellinus igniarius* possessed antioxidant activity *in vitro*, and affected the promotion of cell proliferation, secretion of interleukin (IL)-2, IL-6, and interferon-γ (IFN-γ), and inhibition of tumor necrosis factor-α expression in immune cells. Polysaccharides and flavonoids might enhance immunity in immunocompromised mice and affect the intestinal flora by influencing intestinal short-chain fatty acids.

In conclusion, the articles described here: 1) report the rational use of antibacterial drugs; 2) summarize possible targets and strategies in the discovery of antimicrobial sensitizers to overcome bacterial resistance; and 3) emphasize the importance of drug combinations in overcoming bacterial resistance.
